# Peripheral nerve axonal excitability studies: expanding the neurophysiologist’s armamentarium

**DOI:** 10.1186/s40673-015-0022-2

**Published:** 2015-03-03

**Authors:** William Huynh, Matthew C Kiernan

**Affiliations:** Brain and Mind Research Institute, University of Sydney, Sydney, Australia

**Keywords:** Nerve excitability, Cerebellar ataxia, Cerebellar disorders

## Abstract

Nerve excitability studies have emerged as a recent novel non-invasive technique that offers complementary information to that provided by more conventional nerve conduction studies, the latter which provide only limited indices of peripheral nerve function. Such novel tools allow for the assessment of peripheral axonal biophysical properties that include ion channels, energy-dependent pumps and membrane potential in health and disease. With improvements in technique and development of protocols, a typical study can be completed in a short period of time and rapid measurement of multiple excitability indices can be achieved that provide insight into different aspects of peripheral nerve function. The advent of automated protocols for the assessment of nerve excitability has promoted their use in previous studies investigating disease pathophysiology such as in metabolic, toxic and demyelinating neuropathies, amyotrophic lateral sclerosis, stroke, spinal cord injury and inherited channelopathies. In more recent years, the use of nerve excitability studies have additionally provided insights into the pathophysiological mechanisms underlying cerebellar disorders that include stroke and familial cerebellar ataxias such as episodic ataxia types 1 and 2. Moreover, this technique may have diagnostic and therapeutic implications that may encompass a broader range of neurodegenerative cerebellar ataxias in years to come. In the foreseeable future, this technique may eventually be incorporated into clinical practice expanding the currently available armamentarium to the neurophysiologist.

Conventional nerve conduction studies (NCS) remain an important tool and in many respects, an extension of the clinical assessment in patients with neurological disorders, particularly those pertaining to the peripheral nervous system [1]. However, such techniques that employ supramaximal stimuli to measure amplitude and velocity of compound sensory or motor action potentials, provide information on only the number of conducting fibres and conduction velocity of the fastest, and hence only limited indices of peripheral nerve function.

In more recent years, a novel technique of axonal excitability has emerged and provide complementary information to those offered by conventional NCS [2]. Since the introduction of threshold measurements to study human motor axons in 1970 [3] and its first application in a clinical setting on diabetic patients [4], the technique has undergone modifications and refinement over the years with the development of protocols to allow the rapid measurement of multiple nerve excitability parameters in a short space of time and hence increasing the technique’s practicality when applied in a clinical environment [5].

Axonal excitability techniques provide information related to activity of a variety of ion channels, energy-dependent pumps and ion exchange processes activated during impulse conduction in peripheral axons. While axonal membrane potential cannot be directly measured in intact human axons, indirect evidence may be obtained through assessment of the changes in axonal excitability measured through alterations in current required to elicit an action potential of a defined size [6]. “Threshold” refers to the stimulus current required to produce a predetermined target compound muscle action potential (CMAP) response (e.g., 40% of maximum) and can be that can be adjusted on-line by the computer software (“tracked”) during different manoeuvers (e.g., subthreshold conditioning) to follow changes in nerve excitability [7]. Measurement of threshold depends on and therefore provides an indirect measure of resting membrane potential. Furthermore, resting membrane potential is determined by a complex network of axonal membrane ion channels (persistent Na^+^ channels, slow and fast K^+^ channels) and the activity of the Na^+^/K^+^ pump [8] (Figure 1).

**Figure 1:**
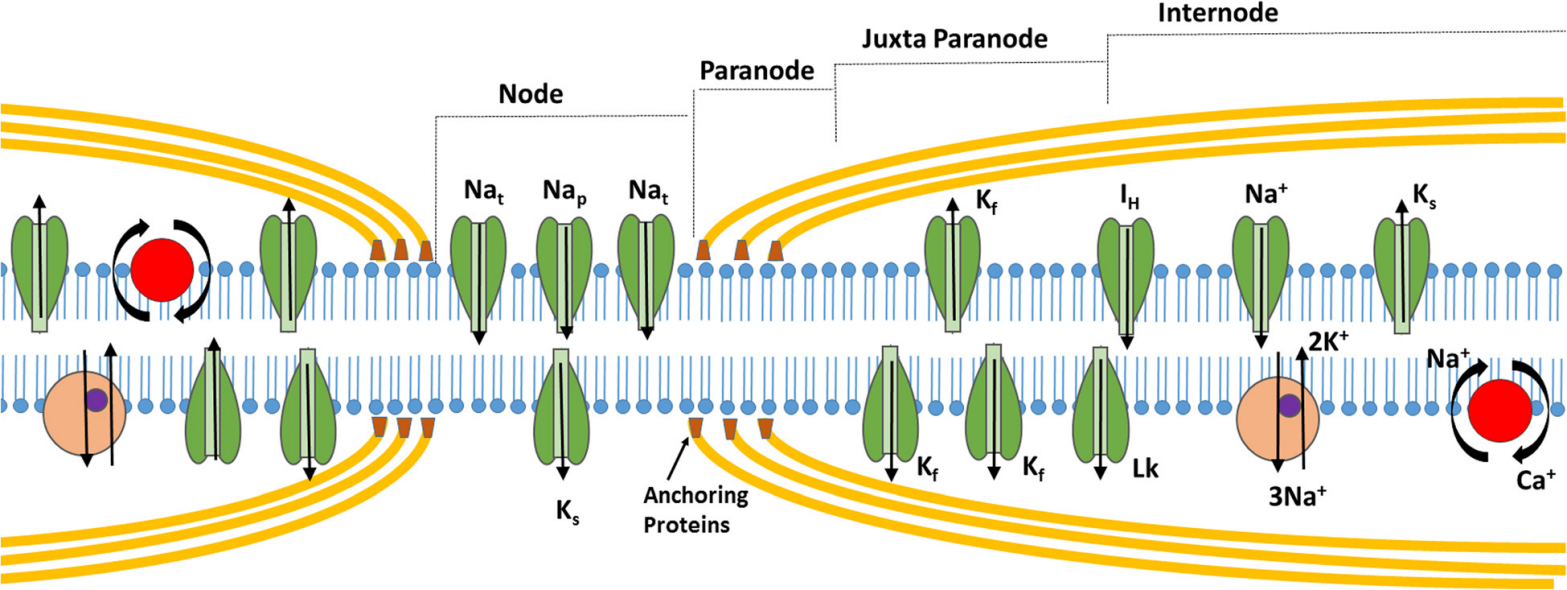
**Diagram of a myelinated axon illustrating ion channels, pumps and exchangers responsible for determining axonal excitability.** Transient Na^+^ channels (Na_t_) are clustered at high density at the node of Ranvier, with persistent Na^+^ channels (Na_p_) and slow K^+^ channels (K_s_) contributing to excitability and resting membrane potential. Fast K^+^ channels (K_f_) are located at highest density at the juxtaparanode, acting to limit re-excitation of the node following an action potential. Internodal conductances include voltage-independent ‘leak’ conductances (Lk) and hyperpolarization-activated cation conductance (I_H_). The Na^+^–K^+^ pump (Na^+^/K^+^-ATPase) utilises energy to maintain the electrochemical gradient necessary for impulse conduction by removing 3 Na^+^ ions for every 2 K^+^ ions pumped into the axon. The Na^+^–Ca2^+^ exchanger exports Ca2^+^ ions and imports Na^+^, driven by the electrochemical Na^+^ gradient. Paranodal myelin terminal loops are depicted with anchoring proteins to form paranodal junctions at the paranodal region.

With the development of the rapid automatic testing protocol known as “TROND” (names after a 3-day training symposium held in 1999 at Trondheim, Norway) and the threshold-tracking software QTRAC (© Institute of Neurology, Queen Square, London, UK) that runs the protocol, a set of axonal excitability indices are generated, that reflect the biophysical properties and membrane potential of the axon [9] (Figure 2).

**Figure 2:**
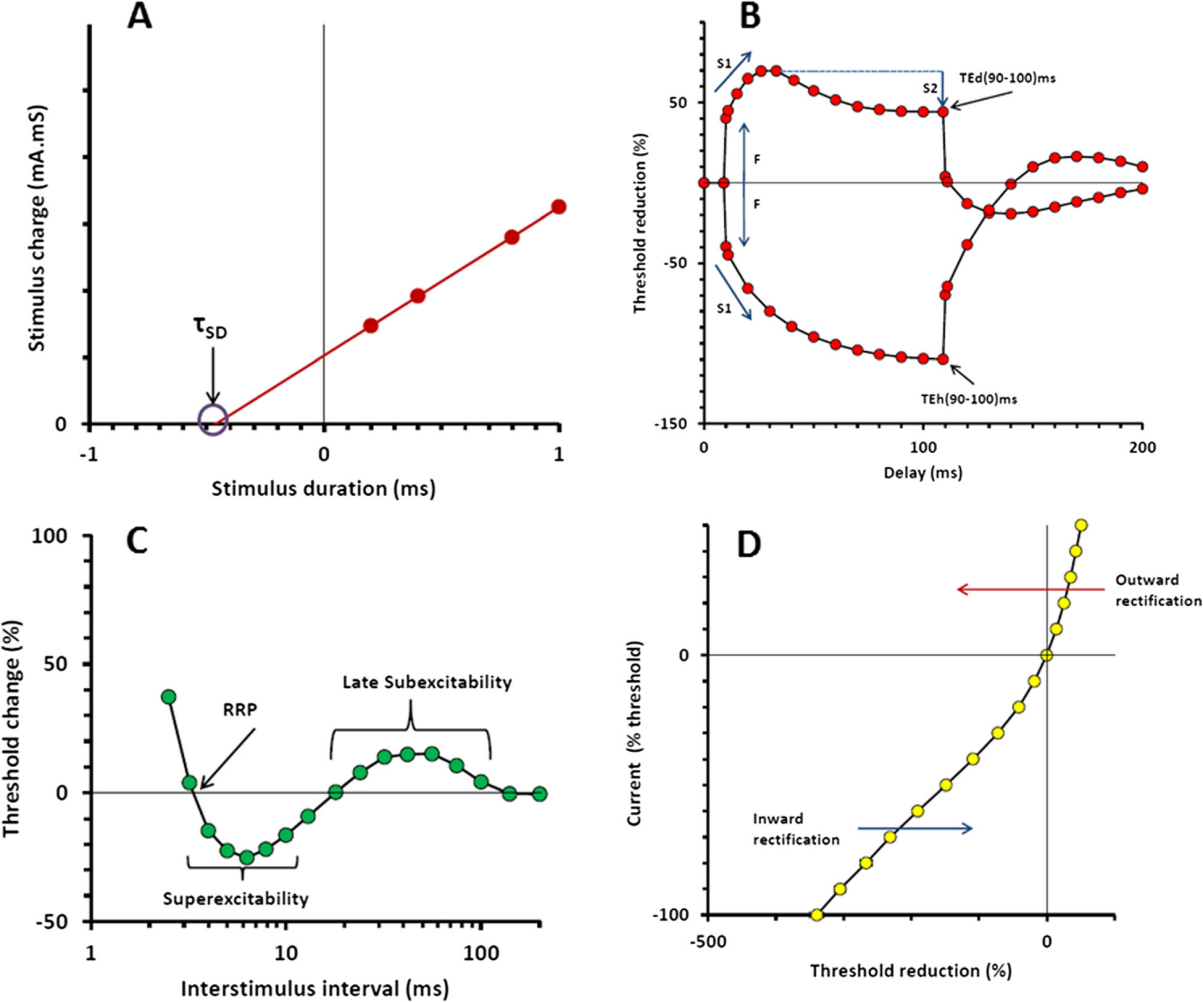
**Plots of excitability parameters recorded from abductor pollicis brevis in a single subject obtained from automated protocol. (A)** Charge-duration relationship, in which intercept on stimulus width axis gives strength-duration time constant and slope gives rheobase. **(B)** Threshold electrotonus for 100 ms polarizing currents, ±40% of threshold. Responses to depolarizing currents start above the line and those to hyperpolarizing currents below the line. **(C)** Recovery cycle following supramaximal stimulation. **(D)** Current-threshold relationship.

These non-invasive techniques allow the assessment of axonal membrane function in vivo in a clinical setting, and provide insight into both normal nerve function and pathophysiological mechanisms in disease [10]. Over the years, nerve excitability studies have been utilized in a diverse range of conditions including toxic, metabolic, both acquired and inherited demyelinating neuropathies, neurodegenerative disorders such as amyotrophic lateral sclerosis, as well as providing insight into pathophysiological changes occurring at the peripheral nerve level in disorders of the central nervous system such as stroke, spinal cord injury and multiple sclerosis [7,11-32]. In more recent years, the use of nerve excitability studies have provided further insights into the pathophysiological mechanisms underlying cerebellar disorders that include stroke and familial cerebellar ataxias such as episodic ataxia types 1 and 2 [33-35]. In addition, this technique may have diagnostic and therapeutic implications that may encompass a broader range of neurodegenerative cerebellar ataxias in years to come.

The peripheral nerve excitability changes observed in ischemic stroke involving the cerebellum may be a reflection of a transynaptic plastic process or alterations in activity of the limb(s) that result from the resultant functional deficit [13,15]. Downstream peripheral nerve excitability changes have been observed in post-stroke patients involving motor pathways in the brain that may reflect alterations in inward rectifying (I_*H*_) and slow K^+^ conductance, suggesting that transynaptic plasticity in peripheral motor axons develop in response to a remote lesion in the central nervous system [14,15], possibly reflecting an alteration or disturbance in supraspinal circuitry and thereby input to spinal motoneurons, consequently resulting in changes in their axonal physiology [11,36]. As such, the biophysical changes present in cerebellar stroke patients may be a consequence of a downstream transynaptic plastic process following changes in excitability reported to occur in both motor cortices in cerebellar stroke [37]. Additionally, the intricate connections that exist between the deep cerebellar nuclei and motoneurons of the cervical spinal cord, may mean that lesions involving these deep cerebellar structures may ultimately affect excitability of downstream lower motor neurons within the circuitry [38-41].

Genetic neuronal channelopathies commonly manifest with paroxysmal symptoms that may vary considerable between patients rendering diagnosis an often challenging feat. In patients with episodic ataxia type 1 (EA1), mutations in KCNA1 gene result in alterations in fast K^+^ channel function. Specifically, the gene encodes for the α subunit of the K_v_1.1 channel. With such channels also present at the juxtaparanodal region of peripheral axons, a specific pattern of nerve excitability abnormalities in patients with EA1 have been observed which do not appear to be different amongst the different mutations [34]. Moreover, there are patients with EA1 who present with a predominantly peripheral phenotype but with typical EA1 changes observed in nerve excitability parameters rendering this a potential useful diagnostic tool in identifying those patients with an atypical presentation [33]. Of further relevance, the changes in parameters in EA1 differ from those seen in acquired autoimmune neuromyotonia (Isaac’s syndrome) which is a channelopathy affecting the same K^+^ channels, suggesting a pathophysiologically different effect on these channels along the axon between the two conditions . Studies on patients with episodic ataxia type 2 (EA2) have also shown a unique pattern of nerve excitability alterations. Mutations of the CACNA1A gene that encodes the Ca_v_2.1 subunit of the voltage-gated Ca^2+^ channels represent the genetic defect underlying this disorder, and the changes observed in axonal function are postulated to have been a result of Ca^2+^ channel dysfunction which consequently affect the function of slow K^+^ channels [35].

Studies have shown changes in voltage-gated K+ channel kinetics present in the cerebellum of murine models with spinocerebellar ataxia type 3 (SCA3) that precede the onset of Purkinje cell loss [42]. Based on these preliminary observations, future studies utilizing nerve excitability in human patients with the spinocerebellar ataxia may allow for the development of a diagnostic electrophysiological biomarker.

Taken together, nerve excitability studies may provide for a sensitive technique that can be applied in a quick and non-invasive manner to facilitate the diagnosis of a range of acquired autoimmune or neurodegenerative as well as genetic cerebellar disorders.

Studies of nerve excitability in chemotherapy-induced neurotoxicity have provided insight into the pathophysiological mechanisms involved and enable early identification of neurotoxicity thereby optimizing treatment strategies and improving patient quality of life in cancer patients [43]. Assessment of motor and sensory nerve function in many chemotherapy-induced neuropathies using conventional NCS has revealed significant reductions in compound sensory action potential (CSAP) amplitude whilst CMAP and conduction velocities are often preserved, consistent with a sensory neuropathy of the axonal type [20,44]. Studies of sensory nerve excitability have demonstrated a direct effect of oxaliplatin on nerve excitability, with changes in sensory axons immediately following infusion similar to those seen with the Na^+^ channel blocker tetrodotoxin [19], suggesting partial blockade of axonal Na^+^ channels [9,20,22]. Longitudinal assessment of axonal excitability have shown that before each successive oxaliplatin treatment, progressive changes in nerve excitability with increased cumulative dosing were observed [45]. There were significant changes in threshold electrotonus and recovery cycle indices. Importantly, progressive changes in sensory nerve excitability across treatment cycles occur before reductions in peak CSAP amplitude are detected [46]. This suggests that such changes may be able to identify at-risk patients prior to the development of chronic neuropathy [47,48].

In diabetic neuropathy patient, studies of nerve excitability have demonstrated alterations in threshold electrotonus consistent with reductions in Na^+^/K^+^ pump function, that subsequently improved following strict glycemic control [23,49]. Other studies have suggested changes in on Na^+^ channel function with alterations in recovery cycle parameters [50]. Recent studies have also shown marked improvement in nerve excitability parameters in patients treated with continuous insulin therapy compared to other regimens [51]. More importantly, changes in nerve excitability preceded the development of neuropathy in diabetic patients thus providing a promising biomarker for detecting preclinical neuropathy in such patients [52,53]. Furthermore, in those patients with typical neuropathic symptoms that may reflect small fibre neuropathy and hence normal results on conventional nerve conduction studies, nerve excitability techniques offer a more sensitive way to establish the presence of altered nerve function underlying these symptoms and providing a potential biomarker to aid the treatment of symptoms in these patients.

Excitability studies in patients with chronic kidney disease and neuropathy have demonstrated significant changes consistent with axonal depolarization driven by hyperkalemia prior to dialysis that normalized following such renal replacement therapies [21,54]. Such techniques have also provided insight into the differential effects of various haemodialysis regimens on nerve function [55], as well as potential neurotoxic effects of various immunosuppressants following renal transplant [56], allowing for the appropriate selection of management strategies involved in renal replacement.

In summary, nerve excitability techniques are a powerful and novel non-invasive means of detecting alterations in axonal biophysical properties that may potentially expand the current armamentarium available to the clinical neurophysiologist. The recent development of commercially available software and hardware represent a step toward implementing these techniques as a clinical diagnostic tool. These measurements are not only important

in investigating the pathophysiology of disorders of the peripheral and to a lesser degree the central nervous systems, they will play a significant role in charting disease progress, and detecting subclinical alterations in nerve function in neuropathies and during treatment with potentially neurotoxic drugs. This in turn will aid in the development of novel therapies for disorders of the nervous system.
